# Analytical Method for Predicting Early Age Thermal Effects in Thick Foundation Slabs

**DOI:** 10.3390/ma12223689

**Published:** 2019-11-08

**Authors:** Barbara Klemczak

**Affiliations:** Faculty of Civil Engineering, Silesian University of Technology, Akademicka 5, 44-100 Gliwice, Poland; Barbara.Klemczak@polsl.pl

**Keywords:** early age concrete, mass concrete, hydration temperature, thermal stress, foundation slabs, analytical method

## Abstract

Prediction of hydration temperature and induced stresses in mass foundation slabs, due to the hydration effects is a difficult task. The complexity of this issue is compounded by transient and non-linear thermo-mechanical phenomena as well by a significant number of contributing technological and material factors that affect the early-age volume changes. This is a probable reason for the limited number of simple analytical methods allowing for the estimation of these effects. This work presents a new proposal in the discussed field. The submitted analytical method for determining the hydration temperature rise, its differentials at a cross-section and induced thermal stresses in mass concrete foundation slabs considers the majority of important technological and material factors, such as the initial temperature of the concrete, the ambient temperature, the thermal properties of the concrete and the heat exchange conditions on the slab surfaces. In stress analysis, both self-balanced and restraint stresses are calculated. Finally, the method is validated in FE analysis conducted for the slabs with various heights and made of different types of cements, as well as by the thermal measurements from the construction site. Due to the limited number of methods allowing for the analytical estimation of the early age thermo-mechanical effects in slabs, this new proposal can be useful in the assessment of these effects.

## 1. Introduction

The main feature of a mass concrete foundation slab at an early age is its thermal volume change due to the hydration heat. These thermal volume changes, due to the hydration temperature rise and subsequent cooling, are accompanied by creep of maturing concrete, which gradually gains the strength and stiffness. Additionally, the described volume changes are restricted by external or internal restraints and, as a consequence, the stresses are induced. 

**Figure 1:**
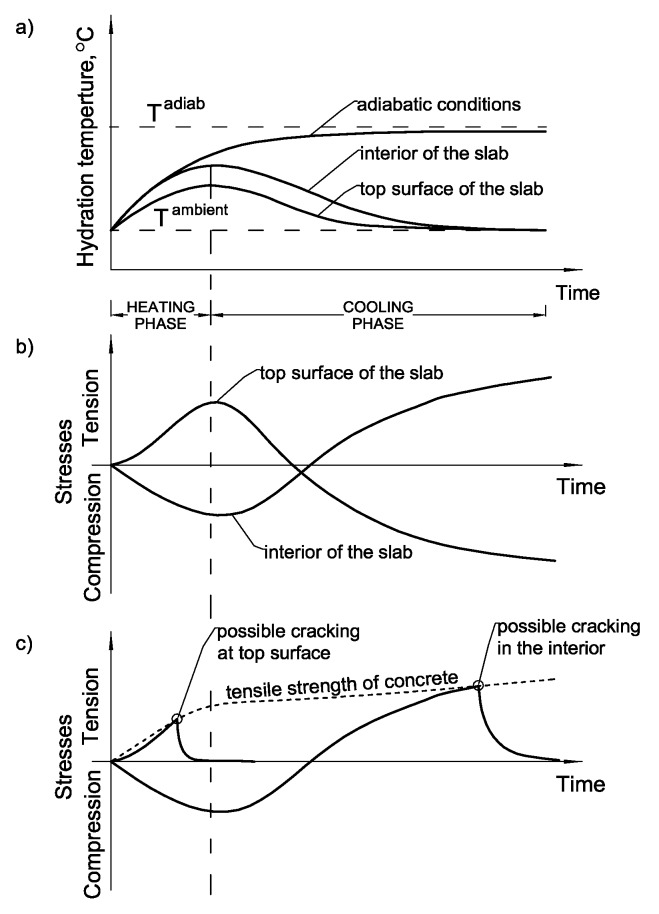
Temperature (**a**), stress (**b**) development and possible cracking (**c**) in mass foundation slab [5].

The external restraints stem from the bond or frictional forces between the foundation slab and the underlaying base. Excepting the foundation on the rock, the stiffness of the subsoil is low in comparison to the concrete and the degree of the external restraints is usually inconsiderable and the stress, due to this effect is not substantial [1]. A significant responsibility for the induced high stresses lies on the internal restraints in mass foundation slabs. The direct cause of the internal restraints in mass slabs are the thermal gradients within the slab thickness [2,3,4]. In this case, the temperature inside the thick slab increases due to the hydration heat and drops very slowly, while the slab surfaces cool quicker, due to the heat transfer to the surroundings (Figure 1a). Thus, the smaller volume changes of the surfaces are restrained by the warmer concrete inside the slab. Such restraint creates the tensile stresses at the slab surfaces in the heating phase, and the cracks can appear when the excessive temperature differences across the slab cross-section arise (Figure 1b). These cracks, of random pattern, are usually observed at the top surface of the slabs within a few days after slab casting (Figure 1c). It should also be added that in the cooling phase, the cracks can also appear inside the slab (Figure 1c). In the cooling phase, the interior of the slab starts to cool down, due to the progressive heat transfer to the environment (Figure 1a), and the inversion of stresses occurs. The previously compressed interior of the slab is subjected to tension and compression arises in surface layers (Figure 1b). However, the construction site experiences show that internal cracks are much less frequent. 

The key to control early age cracking of the described origin is the appropriate prediction of the thermal effects by the designer and transferring the recommendations to the contractor and concrete producer. The prediction of the hydration temperature rise and its gradient, stresses and cracking risk in mass foundation slabs presents particular problems both because of the properties of maturing concrete, as well as the conditions of the construction process. Many parameters are necessary for the analysis discussed above, such as heat evolved during cement hydration, thermal and mechanical properties of hardening concrete and its curing conditions [5,6,7,8,9]. 

The methods used for evaluating the early age thermal effects in mass foundation slabs range from simple analytical methods to complex finite element approaches. Beyond doubt, the finite element analysis provides comprehensive recognition of the problem, including temperature and stress development in the slab in the time of concrete hardening with simultaneous consideration of the material properties and technological conditions. Although not all commercially available software is suitable for this type of analysis, several advanced numerical tools are available to perform the FE analysis of concrete structures at an early age [10,11,12]. The examples of finite element analysis of thermal problems in mass concrete structures can also be found in the scientific literature [13,14,15]. Nevertheless, the use of specialized software is not always accessible for the structural designer. Additionally, the adequate knowledge of thermo-mechanical parameters for hardening concrete often goes beyond the standard engineering knowledge. 

Therefore, the simple analytical methods are still a continued need in this field because of their ease and the possibility of the application without using any specialized software. Despite that some guidelines dedicated to mass concrete recommend the application of numerical methods to design of discussed structures [9,16], the analytical methods can also be found in them [1,8,9] and in the research works [4]. 

Exemplary, British guidelines CIRIA C660 [1] proposed the method to predict values of temperature rise and temperature differentials which is based on standard heat diffusion theory. In this case, the numerical method was implemented to extensive spreadsheet calculations and this way, some flexibility of the analysis is offered [1]. Early-age tensile restrained strains are only calculated at the upper surface of the slab in the heating phase, with the internal restraint factor equal to 0.42, and creep coefficient taken as 0.65 [1]. Next, the latest version of Japanese standards concerning the process of design and reducing the thermal cracking risk in mass concrete structures presents the simplified method resulting from the comprehensive numerical simulations [9]. Following this method, the temperature difference at the slab thickness is not calculated, but the placing temperature, the ambient temperature and the ultimate adiabatic temperature rise are related to the reference values and used in the determination of thermal cracking index. American guidelines ACI 207.2R-07 [8] generally recommends Schmidt’s method for determining temperature gradients at the slab thickness, but also presents some diagrams for adiabatic temperature rise and heat dissipation through mass concrete. Internal restraint factor associated with nonuniform volume change at slab thickness is calculated similarly to the continuous edge restraint. Thus, the general ACI equation for the external restraint factor is used, but with the effective restraining plane taken as a plane of zero stress at the slab thickness. The assumption of this approach is the section stability resulting from the balance between tension at slab surfaces and compression inside the slab, which occurs in the heating phase. 

This work presents a new proposal in the discussed field. A simple analytical method for the determination of hydration temperature rise, temperature gradient and the induced thermal stresses in mass concrete foundation slabs are described. The method has been supported by the finite element analysis of the foundation slabs with various heights and made of different types of cements. In the experimental validation, the temperature measurements in the real foundation slabs have been used. 

## 2. Proposal of the Analytical Method

The proposed method allows for the relatively simple determination of temperature rise, temperature differentials at the slab thickness and stresses in slabs both in the heating and cooling phases of concrete hardening. It should be mentioned that the version of the presented method for temperature evaluation in early age walls has been already presented [17]. For mass foundation slab, the calculation algorithm differs in some coefficients and boundary conditions. The proposed procedure for induced self-balanced thermal stresses is based on the idea of the compensation plane method [18]. The shrinkage strains have been skipped in the proposed procedure, due to their negligible influence on the induced stresses in mass foundation slabs [1,8].

### 2.1. Hydration Temperature Rise and the Temperature Differentials at the Slab Thickness

The adiabatic temperature rise of concrete $\Delta T_{\mathrm{adiab}}$, due to the hydration can be easily calculated from the well-known Equation (1):

(1) $$\Delta T_{\mathrm{adiab}}=\frac{C\cdot Q_{\infty }}{c_{b}\cdot \rho },$$

with C—the mass of cement in 1 m^3^ of concrete, kg; $c_{b}$—specific heat of concrete, kJ/(kg∙°C); ρ—volumetric mass density of concrete, kg/m^3^; $Q_{\infty }$—total heat of cement hydration, kJ/kg. 

Based on the results of own experimental tests [5] the following values of the specific heat are suggested for concrete with different aggregate: Gravel—$c_{b}$ = 0.84 kJ/(kg∙°C); basalt—$c_{b}$ = 0.80 kJ/(kg∙°C); granite—$c_{b}$ = 0.88 kJ/(kg∙°C); limestone—$c_{b}$ = 0.80 kJ/(kg∙°C).

Optionally, the specific heat $c_{b}$ can be taken from the conducted experimental tests or the Equation based on the composition of concrete mix can be used [19]:

(2) $$c_{b}=\sum_{i=1}^{n}m_{i}c_{\mathrm{bi}},$$

where $m_{i}$ is a weight ratio of the subsequent component, in a total mass of 1 m^3^ of concrete, and $c_{\mathrm{bi}}$ is a specific heat for concrete component, given in Table 1. 

Similarly, the total heat of hydration $Q_{\infty }$ can be taken from experiments or calculated from theoretical models considering the different binder constituents. In the presented analytical method, the model proposed by Schindler and Folliard [20] is used. Following this model, Equations (3) and (4) for the total heat of hydration of a binder containing slag, fly ash and silica fume can be used:

(3) $$Q_{\infty }{=p}_{\mathrm{cem}}Q_{\infty }^{\mathrm{cem}}+461p_{\mathrm{slag}}+1800p_{\mathrm{FA}}+330p_{\mathrm{SF}},$$

with $Q_{\infty }^{\mathrm{cem}}$ obtained on the base of Portland cement constituents:

(4) $$Q_{\infty }^{\mathrm{cem}}=500+260p_{c_{2}S}+866p_{c_{3}A}+420p_{c_{4}\mathrm{AF}}+624p_{{\mathrm{SO}}_{3}}+1186p_{\mathrm{freeCaO}}+850p_{\mathrm{MgO}}$$

and $p_{i}$—weight ratio of i-th component in Portland cement; $p_{\mathrm{cem}}$—Portland cement weight ratio; $p_{\mathrm{slag}}$—slag weight ratio; $p_{\mathrm{FA}}$—fly ash weight ratio, $p_{\mathrm{FA}-\mathrm{CaO}}$—CaO weight ratio in terms of total fly ash content; $p_{\mathrm{SF}}$—silica fume weight ratio.

Table 2 presents the total heat of cement hydration calculated with the use of Equations (3) and (4) for various cements, including also those with mineral additives, such as ground granulated blast furnace slag and/or siliceous fly ash. The specified cements have been used later for the numerical validation of the proposed analytical model.

The temperature rise determined in Equation (1) applies to fully adiabatic conditions of concrete curing. In fact, concrete matures in conditions different from adiabatic and a portion of heat is transferred to the environment. The heat transfer to the environmental effects in a lower maximum temperature of concrete than determined in Equation (1), which is for the adiabatic conditions. The proposed two coefficients considering this effect have been derived from FE analysis of foundation slabs with different thickness and made of concrete with various cements. The coefficient $a_{Q}$ represents less heat released due to the occurrence of the maximum temperature in the slab earlier than the time needed to release the total heat of hydration $Q_{\infty }$. The value of the coefficient $a_{Q}$ depends on the cement type, due to the different rate of the heat evolved in the hydration process of cements. Thus, the coefficient $a_{Q}$ was derived on the basis of the results of numerical tests made for adiabatic conditions and different cements. The actual amount of heat released in the first days of hardening was calculated and compared with the adiabatic temperature rise of concrete $\Delta T_{\mathrm{adiab}}$.

The coefficient $a_{d}$ also considers the heat loss from the core of the slab, due to its transfer from surfaces to the environment, but it depends on the thickness of the slab. It is equal to 1 for adiabatic conditions, which can occur in the core of very thick slabs; in another case, it is lower than 1. The coefficient $a_{d}$ decreases in the thinner slabs, because the heat flow from the interior towards the surfaces is faster. Thus, the hardening temperature rise is modified in the following way:

(5) $$\Delta T_{\mathrm{adiab}}^{\mathrm{red}}=a_{Q}\Delta T_{\mathrm{adiab}},$$

and the final temperature in the core of the slab is equal to:

(6) $$T_{\mathrm{int}}=\left(T_{o}+\Delta T_{\mathrm{adiab}}^{\mathrm{red}}\right)a_{d}.$$

with the initial temperature of concrete $T_{o}$. The suggested values of coefficients are visible in Table 3 and Table 4.

Temperatures at the surfaces of the slab are determined on the base of 3^rd^ type boundary condition, which is known in general form as:

(7) $${\frac{\mathrm{dT}}{\mathrm{dx}}|}_{\mathrm{st}}=-\frac{\alpha_{p}}{\lambda },\left(T_{s}-T_{a}\right),$$

with λ—thermal conductivity of concrete, W/(m∙°C); $\alpha_{p}$—heat transfer coefficient, W/(m^2^·°C); $T_{s}$—surface temperature, °C; $T_{a}$—outside temperature, °C.

**Figure 2:**
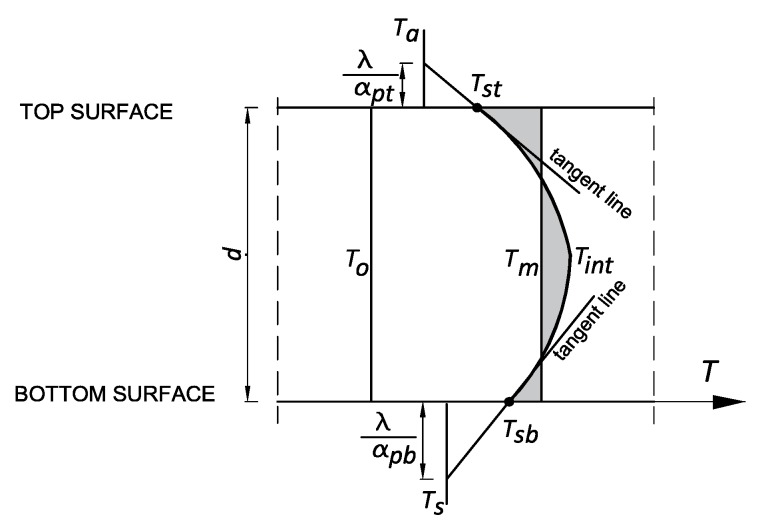
Distribution of temperature at the thickness of the slab.

Available test results of temperature distribution at the thickness of massive members show that the temperature profile can be approximated by a parabola, as shown in Figure 2 [1,21]. Thus, assuming parabolic distribution of temperature at the thickness of the slab and the diversified coefficients of heat transfer for top surface $\alpha_{\mathrm{pt}}$ and bottom surface $\alpha_{\mathrm{pb}}$, the precise Equations have been derived:

- for the top surface of the slab:
  (8) $$T_{\mathrm{st}}=T_{\mathrm{int}}+0.5d\frac{T_{a}-T_{\mathrm{int}}}{0.5d+2\frac{\lambda }{\alpha_{\mathrm{pt}}}},$$
- for the bottom surface of the slab:
  (9) $$T_{\mathrm{sb}}=T_{\mathrm{int}}+0.5d\frac{T_{s}-T_{\mathrm{int}}}{0.5d+2\frac{\lambda }{\alpha_{\mathrm{pb}}}}.$$

Similarly as for the specific heat, considering the results of own experimental tests [5], the following values are suggested for concrete with different aggregate: Gravel—λ = 2.96 W/(m∙°C); basalt—λ = 2.04 W/(m∙°C); granite—λ = 2.41 W/(m∙°C); limestone—λ = 2.48 W/(m∙°C).

It is also possible to take thermal conductivity λ from the conducted experimental tests or from the theoretical formula [19]:

(10) $$\lambda =\sum_{i=1}^{n}m_{i}\lambda_{I},$$

where $m_{i}$ is a weight ratio of the subsequent component, in a total mass of 1 m^3^ of concrete, and $\lambda_{i}$ is a thermal conductivity for concrete component, given in Table 5. 

Coefficients of heat transfer are crucial in the evaluation of temperature at slab surfaces. The coefficient $\alpha_{\mathrm{pt}}$ represents the heat exchange between the concrete and the surrounding environment, which occurs due to convection or radiation. In prediction models, one value of the coefficient is usually assumed, additionally dependent on wind speed. The values of $\alpha_{\mathrm{pt}}$ for the top surface of the slab can be taken from Table 6 [19].

If any insulation layer is applied on the slab top surface, the coefficient $\alpha_{\mathrm{pt}}$ should be modified according to the well-known formula [19]:

(11) $$\alpha_{{\mathrm{pt}}_{\mathrm{mod}}}=\frac{\alpha_{\mathrm{pt}}\lambda_{i}}{d_{i}\alpha_{\mathrm{pt}}+\lambda_{i}},$$

with $\lambda_{i}$—thermal conductivity coefficient of the insulation layer, W/(m·°C), and d_i_—thickness of the insulation layer, m. For the bottom surface of the slab, based on the performed numerical analysis, the recommended value is $\alpha_{\mathrm{pb}}$ = 3.0 W/(m^2^·°C).

Finally, the mean temperature has been calculated for the parabolic distribution of temperature at the slab thickness (Figure 2):

(12) $$T_{m}=\frac{2}{3}T_{\mathrm{int}}+\frac{1}{6}\left(T_{\mathrm{st}}+T_{\mathrm{sb}}\right)$$

### 2.2. Thermal Strains and Stresses

**Figure 3:**
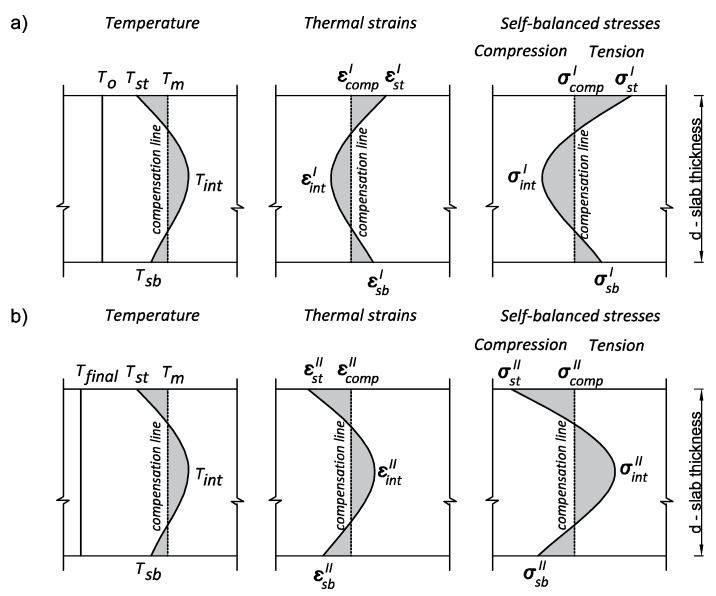
The idea of the compensation plane method – distribution of temperature, thermal strains and stresses at the thickness of the slab: (**a**) Heating phase, (**b**) cooling phase [9].

The induced strains and stresses, due to the internal restraints (caused by the temperature difference between the core of the slab and its surfaces) have been determined using the compensation plane method [18]. The idea of this method is presented in Figure 3. It was assumed in the proposed model that in case of the dominant internal restraint, the stress distribution has the same shape through the cross-section as the temperature profile. Consequently, it has been assumed that the line representing the mean temperature at the slab thickness is also a line of zero stresses.

As the self-balanced stresses are caused by temperature gradients at the thickness of the slab, they have been determined on the basis of the difference between the actual strain and the strain on the compensation line (Figure 3). Therefore, in the heating phase (denoted with the superscript ‘I’), and in the cooling phase (denoted with the superscript ‘II’), the Equations (13)–(18) can be used to calculate the stresses at three crucial points at the slab thickness (Figure 3):

- In the center of the slab:
  (13) $$\sigma_{\mathrm{int}}^{I}=E_{\mathrm{cm},\mathrm{eff}}^{I}\left(\varepsilon_{\mathrm{comp}}^{I}-\varepsilon_{\mathrm{int}}^{I}\right)-\;\mathrm{heating}\;\mathrm{phase},$$
  (14) $$\sigma_{\mathrm{int}}^{\mathrm{II}}=E_{\mathrm{cm},\mathrm{eff}}^{\mathrm{II}}\left(\varepsilon_{\mathrm{comp}}^{\mathrm{II}}-\varepsilon_{\mathrm{int}}^{\mathrm{II}}\right)-\;\mathrm{cooling}\;\mathrm{phase},$$
- At the top surface of the slab:
  (15) $$\sigma_{\mathrm{st}}^{I}\;=E_{\mathrm{cm},\mathrm{eff}}^{I}\left(\varepsilon_{\mathrm{comp}}^{I}-\varepsilon_{\mathrm{st}}^{I}\right)-\;\mathrm{heating}\;\mathrm{phase},$$
  (16) $$\sigma_{\mathrm{st}}^{\mathrm{II}}\;=E_{\mathrm{cm},\mathrm{eff}}^{\mathrm{II}}\left(\varepsilon_{\mathrm{comp}}^{\mathrm{II}}-\varepsilon_{\mathrm{st}}^{\mathrm{II}}\right)-\;\mathrm{cooling}\;\mathrm{phase},$$
- At the bottom surface of the slab:
  (17) $$\sigma_{\mathrm{sb}}^{I}\;=E_{\mathrm{cm},\mathrm{eff}}^{I}\left(\varepsilon_{\mathrm{comp}}^{I}-\varepsilon_{\mathrm{sb}}^{I}\right)-\;\mathrm{heating}\;\mathrm{phase},$$
  (18) $$\sigma_{\mathrm{sb}}^{\mathrm{II}}\;=E_{\mathrm{cm},\mathrm{eff}}^{\mathrm{II}}\left(\varepsilon_{\mathrm{comp}}^{\mathrm{II}}-\varepsilon_{\mathrm{sb}}^{\mathrm{II}}\right)-\;\mathrm{cooling}\;\mathrm{phase}.$$

The strains included in Equations (13)–(18) are determined using the following formulas:

- Strains on the compensation line:
  (19) $$\varepsilon_{\mathrm{comp}}^{I}\;=\alpha_{T}\left(T_{m}-T_{o}\right)-\;\mathrm{heating}\;\mathrm{phase},$$
  (20) $$\varepsilon_{\mathrm{comp}}^{\mathrm{II}}\;=\alpha_{T}\left(T_{\mathrm{final}}-T_{m}\right)-\;\mathrm{cooling}\;\mathrm{phase},$$
- Strains in the center of the slab:
  (21) $$\varepsilon_{\mathrm{int}}^{I}\;=\alpha_{T}\left(T_{\mathrm{int}}-T_{o}\right)-\;\mathrm{heating}\;\mathrm{phase},$$
  (22) $$\varepsilon_{\mathrm{int}}^{\mathrm{II}}\;=\alpha_{T}\left(T_{\mathrm{final}}-T_{\mathrm{int}}\right)-\;\mathrm{cooling}\;\mathrm{phase},$$
- Strains at the top surface of the slab:
  (23) $$\varepsilon_{\mathrm{st}}^{I}\;=\alpha_{T}\left(T_{\mathrm{st}}-T_{o}\right)-\;\mathrm{heating}\;\mathrm{phase},$$
  (24) $$\varepsilon_{\mathrm{st}}^{\mathrm{II}}\;=\alpha_{T}\left(T_{\mathrm{final}}-T_{\mathrm{st}}\right)-\;\mathrm{cooling}\;\mathrm{phase},$$
- Strains at the bottom surface of the slab:
  (25) $$\varepsilon_{\mathrm{sb}}^{I}\;=\alpha_{T}\left(T_{\mathrm{sb}}-T_{o}\right)-\;\mathrm{heating}\;\mathrm{phase},$$
  (26) $$\varepsilon_{\mathrm{sb}}^{\mathrm{II}}\;=\alpha_{T}\left(T_{\mathrm{final}}-T_{\mathrm{sb}}\right)-\;\mathrm{cooling}\;\mathrm{phase},$$

The recommended values of the coefficient of thermal expansion $\alpha_{T}$ for a different type of aggregate in concrete are taken from [1] and listed in Table 7.

For calculations of induced stresses described by Equations (13)–(18), a development of the modulus of elasticity during concrete hardening is required. In the proposed method, the recommendations of Model Code 90 [22] are used for this purpose:

(27) $$E_{\mathrm{cm}}\left(t\right)=E_{\mathrm{cm}}\sqrt{e^{s{\left(1-\left(\frac{28}{t}\right)\right)}^{0.5}}}$$

with t—age of concrete in days; E_cm_—a mean value of the modulus of elasticity of 28-day concrete in MPa, s—a coefficient dependent on the type of cement. 

The creep is considered by means of the effective modulus of elasticity:

(28) $$E_{\mathrm{cm},\mathrm{eff}}\left(t\right)=\frac{E_{\mathrm{cm}}\left(t\right)}{1+\varphi \left(t,t_{o}\right)}$$

where $\varphi \left(t,t_{o}\right)$ is a creep coefficient. The suggested value of this coefficient for the heating phase is 1.1, based on the results of the numerical analysis and [1,23], where it has been suggested that in an early age concrete with hydration temperature affects the stresses may be reduced about 50%. 

This part of the model requires special attention because of the concrete age corresponding to the occurrence of the maximum hardening temperature and the age at which the cooling of the slab will be completed. The age of concrete for which the slab will be completely cooled is relatively easy to determine—it can be assumed that it will be at least 28-day concrete. For the heating phase, the time of the maximum temperature occurrence is intractable to define. Thus, in the presented procedure, the time t depends on the slab thickness (Table 8). Generally, this time is greater than the actual time when the maximum temperature occurs (usually 2–5 days) because it also considers accelerated hardening of concrete in the conditions of the elevated temperature, which occurs in mass concrete members. Values listed in Table 8 were estimated using the results of finite element analysis (described in Section 3).

Although internal restraints resulting from temperature gradients within the slab and induced self-balanced stresses are dominant, the stresses induced by the external restraints are also considered:

(29) $$\sigma_{R}^{I}=E_{\mathrm{cm},\mathrm{eff}}^{I}\left(\alpha_{T}{\Delta T}^{I}\right)R\;–\;\mathrm{heating}\;\mathrm{phase},$$

(30) $$\sigma_{R}^{\mathrm{II}}=E_{\mathrm{cm},\mathrm{eff}}^{\mathrm{II}}\left(\alpha_{T}{\Delta T}^{\mathrm{II}}\right)R\;–\;\mathrm{cooling}\;\mathrm{phase},$$

with

(31) $${\Delta T}^{I}=T_{m}-T_{0}\;–\;\mathrm{average}\;\mathrm{temperature}\;\mathrm{rise}\;\mathrm{in}\;\mathrm{the}\;\mathrm{heating}\;\mathrm{phase},$$

(32) $${\Delta T}^{\mathrm{II}}=T_{\mathrm{final}}-T_{m}\;–\;\mathrm{average}\;\mathrm{temperature}\;\mathrm{drop}\;\mathrm{in}\;\mathrm{the}\;\mathrm{cooling}\;\mathrm{phase}.$$

**Figure 4:**
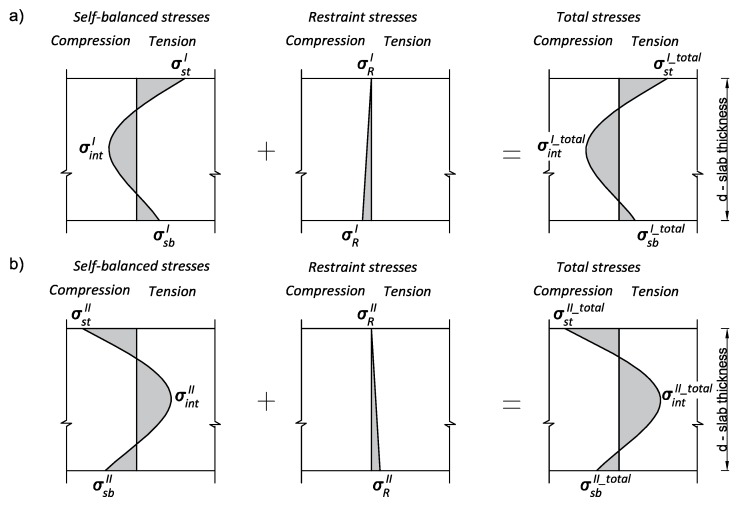
Total thermal stresses at the thickness of the slab: (**a**) Heating phase, (**b**) cooling phase.

The recommended value of the external restraint coefficient R for mass slabs founded on the soil of the medium stiffness is 0.1 at the bottom and 0 at the top of the slab [1]. Finally, it should be formally stated that the total stress is a sum of the self-balanced and externally induced stresses (Figure 4). It should also be mentioned that external restraint thermal stresses have a different character than self-balanced stresses. In the phase of temperature increase (heating phase), the slab is subjected to compression. This indicates that during the heating phase, the restraint stresses reduce tensile stresses induced, due to the internal restraints at the top and bottom surface of the slab. In the cooling phase, external restraints produce tensile stress in the slab and enlarge tensile stresses inside the slab (Figure 4).

## 3. Numerical Validation 

### 3.1. Data for Analysis

Numerical validation of the presented analytical model was performed with the use of TEMWIL and MAFEM original software [24,25]. This software allows for 3D FE analysis of the behavior of massive foundation slabs in the early stages of concrete hardening. The temperature field is determined on the base of the partial differential equation for energy balance, while for the stress analysis, the viscoelastic material model of ageing concrete is applied. Therefore, the FE analysis of a mass slab consists of two steps. The first step is related to the determination of temperature and the thermal strains (TEMWIL). Next, these strains are used as an input for computation of stress development in the second step (MAFEM). 

The slabs with the thicknesses of 1 m, 2 m, 3 m and 4 m were analyzed. The dimensions in the plan view were 20 m × 20 m. In all cases the same concrete mix composition was assumed, only the type of cement was diversified. Concrete mix composition was, as follows—300 kg/m^3^ of cement, 150 l/m^3^ of water, 583 kg/m^3^ of 0–2 mm sand, 427 kg/m^3^ of 2–8 mm aggregate, 389 kg/m^3^ of 8–16 mm aggregate and finally 544 kg/m^3^ of 16–31.5 mm aggregate. Six types of cement were applied in the concrete mix. These cements included both ordinary Portland cement (CEM I), ground-granulated blast furnace slag cements (CEM II/BS and CEM III), siliceous fly ash cements (CEM II/BV), as well as cements with both slag and fly ash (CEM V and VLH). Compositions of all considered cements were listed in chapter 2.1. Detailed thermal and mechanical properties are listed in Table 9. These properties were acquired from the conducted experimental tests [5,17].

**Figure 5:**
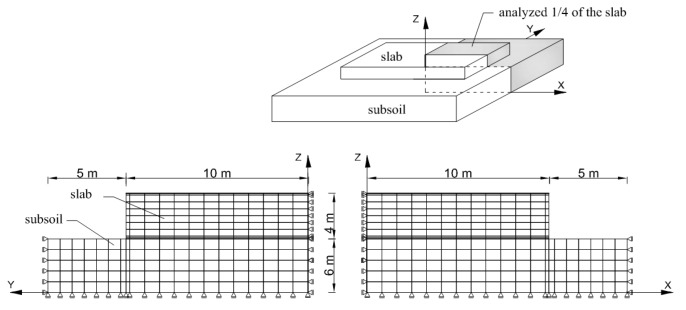
Finite element mesh for the exemplary slab.

The remaining data necessary for analysis are presented in Table 10. Figure 5 shows the finite element mesh and support conditions for ¼ of the analyzed foundation slabs and cooperating soil. For soil, the modulus of elasticity was assumed as equal to 50 MPa, friction angle equal to 30 and zero cohesion.

### 3.2. Results of Validation

**Figure 6:**
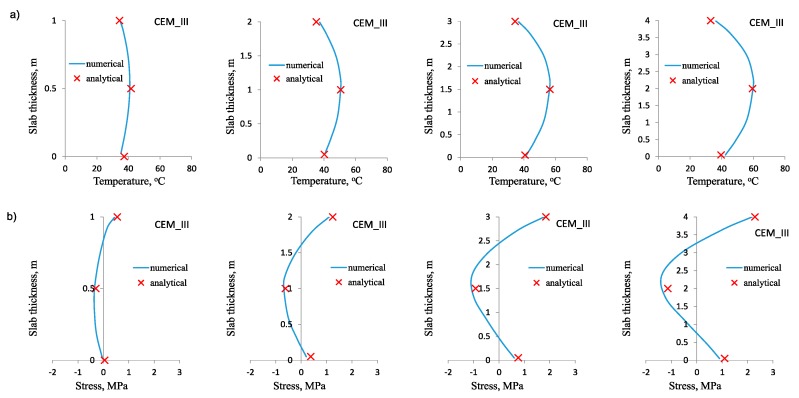
Numerical and analytical results for slabs made of concrete with cement CEM III: (**a**) Temperature distribution at the slab thickness, (**b**) total stress distribution at the slab thickness.

**Figure 7:**
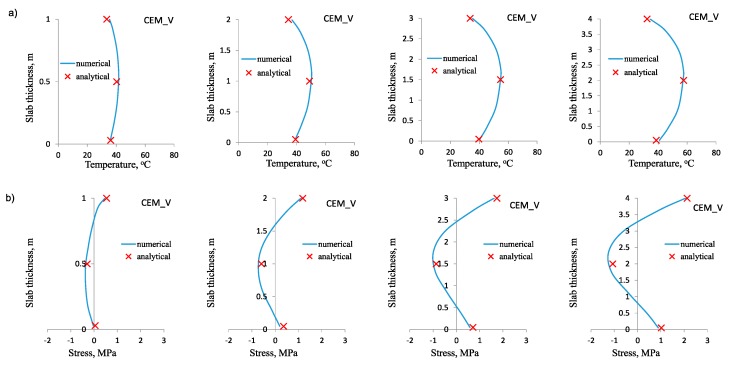
Numerical and analytical results for slabs made of concrete with cement CEM V: (**a**) Temperature distribution at the slab thickness, (**b**) total stress distribution at the slab thickness.

**Figure 8:**
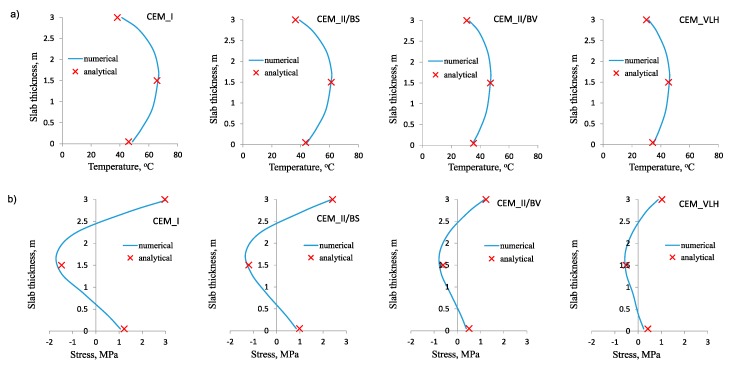
Numerical and analytical results for slabs made of concrete with different cements: (**a**) Temperature distribution at the slab thickness, (**b**) total stress distribution at the slab thickness.

The results of the calculations are shown in Figure 6, Figure 7 and Figure 8. Diagrams of temperature and stress distribution refer to the vertical cross-section of the slab located in the intersection of its vertical planes of symmetry. The maximum values of temperature and stress in the heating phase are depicted as particularly important, due to the possible cracking risk at the top surface of the slab. 

In all cases, the numerical and analytical results obtained for temperature distribution are practically indistinguishable (Figure 6a, Figure 7a, Figure 8a). It was true for the slabs of different thickness and made of concrete with different cements. This compatibility confirms the proper assumption of the model coefficients in thermal analysis. Therefore, this part of the analytical model can also be used independently for determination of temperature rise and its differentials at the thickness of the slab even if the stress analysis is not performed or an alternative analytical model is used for stress evaluation. At this point, the easiness of using the proposed method for slabs in comparison with the Schmidt’s method [8] or the method proposed by CIRIA C660 [1] can be highlighted.

Similarly, the good agreement between the numerical and analytical results was obtained for stress distribution in the heating phase (Figure 6b, Figure 7b, Figure 8b). This conclusion is also valid for all analyzed slabs. The only small discrepancies occurred in the core of the slabs, where the compressive stresses are induced. Simultaneously, this slight incompatibility of the analytical model does not refer to the value of the maximum tensile stress at the top surface, which is crucial for the cracking risk assessment. It has been also noted that the proper estimation of early age stresses induced in the foundation slab is more bothering than estimating temperature changes. The main reason is the difficulties with the determination of the proper value of the modulus of elasticity in the heating phase, as well as creep coefficient of concrete. In the analytical method, it was proposed that the development of the modulus of elasticity depends not only on the concrete age, but also on the thermal conditions in the slab. Thus, the proper value of the modulus of elasticity is calculated for 3, 4, 5 and 6 days of concrete curing for the slabs with the thickness of 1 m, 2 m, 3 m and 4 m respectively. The assumed time considers both concrete age and the accelerated development of the modulus of elasticity, due to the diversified temperature rise in the slab with different thickness. Undoubtedly, this part of the analytical model with the proposed strict values of time is the simplified approach. Nevertheless, the obtained results showed no significant inconsistency. Similar doubts may be raised regarding the value of creep coefficient, which is proposed to be equal to 1.1 for all cement types in concrete. Nevertheless, good agreement was achieved between the numerical and analytical results.

## 4. Experimental Validation 

**Figure 9:**
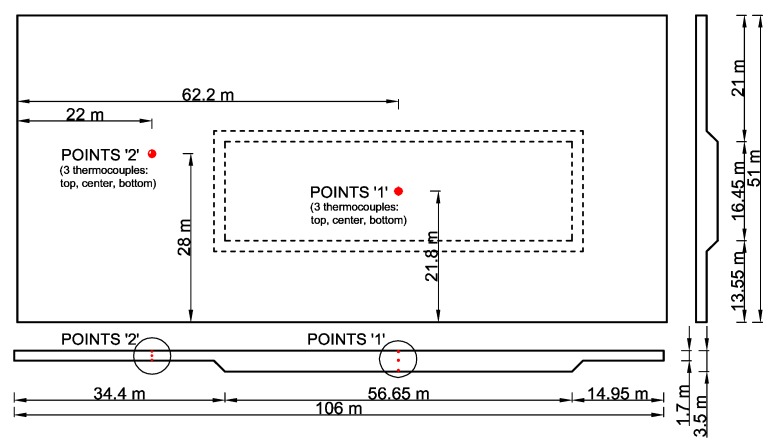
The dimension of the foundation slab with the position of the thermocouples in points ‘1’ and ‘2’.

**Figure 10:**
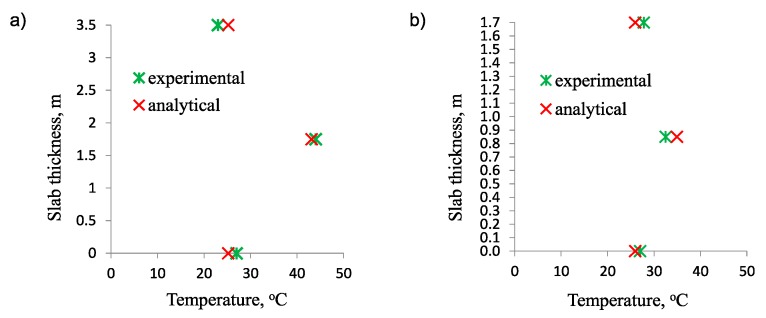
Comparison of experimental and analytical results for the foundation slab: (**a**) Points ‘1’, (**b**) points ‘2’.

The experimental validation of the proposed analytical model has been limited to the thermal analysis. This limitation was dictated by the unavailability of stress measurements in the available examples of the real slabs. Data from one foundation of the power station was used for thermal analysis [26]. The dimension of the slab in a plan view is 106 m × 51 m, while the thickness changes from 1.7 m to 3.5 m (Figure 9). The comparison has been made based on the temperature change recorded by the thermocouples in point ‘1’ and ‘2’ (Figure 9). All necessary data for thermal analysis is listed in Table 11. The results of the comparison between the measured temperature distribution and the values obtained from the analytical model are visible in Figure 10. As in the case of numerical validation, quite good consistency of results is visible. 

## 5. Conclusions 

Prediction of the temperature rise and induced stresses in mass foundation slabs, due to the hydration effects is a particularly difficult challenge. The complexity of this issue is compounded by the non-stationarity and non-linearity of the thermo-mechanical phenomena as well by a significant number of contributing technological and material factors influencing the early-age volume changes. This is the probable reason for a limited number of simple methods allowing for the analytical estimation of these effects. 

In the article, a new analytical method for prediction of thermo-mechanical behavior in mass foundation slab is presented. The detailed procedure with all necessary coefficients for the hydration temperature rise, its differentials at a cross-section of the slab, as well as for the induced thermal stresses are described. The method for determining thermal loads related to the hydration process considers the majority of important factors, such as the initial temperature of the concrete, the ambient temperature, the thermal properties of the concrete as specific heat and thermal conductivity, as well as the heat exchange conditions on the slab surfaces. Additionally, the method seems to be simpler than methods based on standard heat diffusion theory or Schmidt’s method. In the mechanical part of the model, both self-balanced and restraint stresses are calculated. The applied procedure proposed is based on the compensation plane method. Finally, the correctness of the proposed method is confirmed by the results of a finite element analysis of the exemplary slabs and the thermal measurements from the construction site. Nevertheless, no theoretical model is perfect and can always be improved. In this respect, the model coefficients and assumptions should be further tested and possibly clarified, especially in relation to the mechanical properties of concretes made of cements with non-clinker constituents [27,28]. It should also be mentioned that this simple analytical method provides rough estimation resulting from the assumed simplifications. Nevertheless, it can be considered at least as a method of a preliminary analysis of the foundation slab subjected to thermo-mechanical effects. 

## Figures and Tables

**Table 1:** Specific heat $c_{\mathrm{bi}}$ for concrete components, on the basis of [19].

Component	$c_{bi}$, kJ/(kg °C)
water	4.18
cement	0.56
sand	0.74
basalt	0.77
dolomite	0.82
granite	0.47
quartz	0.72
riolite	0.78

**Table 2:** Total heat of cement hydration calculated based on Equation (3).

Cement Type	Component, %			$Q_{\infty }$
	Portland Clinker	Slag (S)	Siliceous Fly Ash (V)	kJ/kg
CEM I 42.5R	95.7	–	–	501
CEM II/B-V 32.5R	67.3	–	29.1	410
CEM II/B-S 32.5R	68.3	27.1	–	490
CEM III/A 32.5N-LH/HSR/NA	41.1	58.9	–	498
CEM V/A (S-V) 32.5R-LH	62.2	18.2	19.6	430
VLH V/B (S-V) 22.5	32.3	34.4	33.3	362

**Table 3:** Reduction coefficient $a_{Q}$.

Cement Type	$a_{Q}$
CEM I 42.5R	0.65
CEM II/B-V 32.5R	0.48
CEM II/B-S 32.5R	0.60
CEM III/A 32.5N-LH/HSR/NA	0.52
CEM V/A (S-V) 32.5R-LH	0.58
VLH V/B (S-V) 22.5	0.50

**Table 4:** Reduction coefficients $a_{d}$.

Slab Thickness, m	$a_{d}$
1.0	0.70
2.0	0.85
3.0	0.95
≥ 4.0	1.0

**Table 5:** Thermal conductivity $\lambda_{i}$ for concrete components, on the basis of [19].

Component	$\lambda_{i}$, W/(m∙°C)
water	0.60
cement	1.28
sand	3.08
basalt	1.91
dolomite	4.32
granite	2.94
quartz	4.60
riolite	1.88

**Table 6:** Coefficient of heat exchange depending on the wind speed [19].

Wind Speed [m/s]	0	1	2	3	4	5	6
$\alpha_{\mathrm{pt}}$ [W/(m^2^·°C)]	6.0	10.4	14.5	18.6	22.6	26.7	34.5

**Table 7:** The coefficient of thermal expansion of concrete [1].

Coarse Aggregate Applied in Concrete	$\alpha_{T}$ $,\;10^{-6}/{}^{\circ}C$
Basalt	10
Flint gravel	12
Quartzite	14
Granite	10
Limestone	9
Sandstone	12.5

**Table 8:** The suggested age of concrete for the estimation of the modulus of elasticity $E_{\mathrm{cm}}\left(t\right).$

Slab Thickness, m	t, Days
1.0	3
2.0	4
3.0	5
≥ 4.0	6

**Table 9:** Thermo–mechanical properties of concretes used in the FE analysis and analytical calculation.

Symbol	Cement	Aggregate	λ	$c_{b}$	ρ	$E_{\mathrm{cm}}$
			W/m °C	kJ/(kg °C)	kg/m^3^	MPa
CEM I	CEM I 42.5R	gravel	2.96	0.84	2370	36400
CEM II/BS	CEM II/B-S 32.5R	gravel	2.96	0.84	2370	33900
CEM II/BV	CEM II/B-V 32.5R	gravel	2.96	0.84	2366	26200
CEM III	CEM III/A 32.5N-LH/HSR/NA	gravel	2.96	0.84	2343	32100
CEM V	CEM V/A (S-V) 32.5R-LH	gravel	2.96	0.84	2366	28800
CEM VLH	VLH V/B (S-V) 22.5	gravel	2.96	0.84	2330	25700

**Table 10:** Coefficients used in numerical and analytical calculations.

Coefficient	Notation	Value	Comment
Initial temperature	$T_{o}$, °C	20	–
Ambient, soil temperature	$T_{a},\;T_{s}$, °C	20	–
Coefficient of thermal diffusion	$\alpha_{\mathrm{TT}}$, m^2^/s	–	calculated: $\alpha_{\mathrm{TT}}=\lambda /\left({\rho c}_{b}\right)$
Rate of hydration heat	$q_{v},$ W/m^3^	–	based on Table 1 and [5]
Thermal transfer coefficient	$\alpha_{p}$, W/(m^2^°C)	6.0 3.5 3.0	top surface side surfaces bottom surface
Coefficient of thermal expansion	$\alpha_{T}$, 1/°C	10 × 10^−6^	–
Coefficient of mechanical development	s	0.2 0.25 0.25 0.38 0.25 0.38	CEM I 42.5R CEM II/B-S 32.5R CEM II/B-V 32.5R CEM III/A 32.5N-LH/HSR/NA CEM V/A (S-V) 32.5R-LH VLH V/B (S-V) 22.5

**Table 11:** Data for the experimental validation of the analytical model.

Data	Unit	Value	Comment
Cement CEM III/A 32.5N-LH/HSR/NA	kg/m^3^	235	The composition of the binder is close to the cement VLH (28% of Portland clinker, 40% of slag and 32% of fly ash in the total amount C= 345 kg/m^3^ of the binder), thus the coefficients were taken as for this cement with C= 345 kg/m^3^ in Equation (1)
Fly ash	kg/m^3^	110	
Density, ρ	kg/m^3^	2306	On the base of experimental data
Specific heat, $c_{b}$	kJ/(kg °C)	0.84	Gravel aggregate
Thermal conductivity, λ	W/(m °C)	2.96	Gravel aggregate
Initial temperature, $T_{o}$	°C	12	On the base of experimental data
Ambient, soil temperature, $T_{a},\;T_{s}$	°C	5	On the base of experimental data
Thermal transfer coefficient, $\alpha_{p}$	W/(m^2^ °C)	3.0 3.0	top surface (with insulation) bottom surface
